# Socioeconomic inequality in psychological distress among older adults in India: a decomposition analysis

**DOI:** 10.1186/s12888-021-03192-4

**Published:** 2021-04-07

**Authors:** Shobhit Srivastava, Naina Purkayastha, Himanshu Chaurasia, T. Muhammad

**Affiliations:** 1 International Institute for Population Sciences, Mumbai, Maharashtra 400088 India; 2 Department of Statistics, Dibrugarh University, Dibrugarh, Assam India; 3 National Institute for Research in Reproductive Health, ICMR, Mumbai, 400088 India

**Keywords:** Socio-economic condition, Health status, Psychological distress, Concentration index, Decomposition analysis

## Abstract

**Background:**

Older people coming from a lower wealth gradient are more vulnerable to have stressful life events further adding more risk for common mental health disorders and psychological distress situations. The present study explores the associations between socioeconomic and health-related variables and psychological distress among older adults in India and the contribution of such factors to the inequalities in psychological distress.

**Methods:**

A cross-sectional survey of 9181 older adults conducted as ‘Building a Knowledge Base on Population Ageing in India’ was assessed. Logistic regression and decomposition models were used to analyze the data. Psychological distress was measured from General Health Questionnaire (GHQ-12). The value of Cronbach's alpha was 0.90. It was having a scale of 0 to 12 on the basis of experiencing stressful symptoms and was re-coded as 0 (representing 6+ stressful symptoms) and 1 (representing 5 and fewer symptoms).

**Results:**

Older adults from the poored, suffering from multi-morbidity, disabled, with low activities of daily living and low instrumental activities of daily living and poor cognitive ability were suffering from high psychological distress in India. Further, factors such as religion, caste, education, living arrangements, and self-worth in the family were major contributors to the concentration of psychological distress in older adults from poor households (concentration index: − 0.23).

**Conclusion:**

The study suggests that among older people, there is a wide disparity of experiencing psychological distress across different socio-economic groups with significant factors being responsible for inequality in psychological distress. There is a need to build a “win-win” circumstance across sectors, including a broad spectrum of health, social and economic benefits to the vulnerable older population.

## Introduction

A considerable increase in life expectancy with the advancement of public health and medical facilities has shifted the population age structure [1]*,* including India where the profile of older adults has changed to 103.8 million (8.6% of the population) in 2011, from 19.6 million (5% of the total population) in 1951 [2]. Again, the global estimates of the greying population are expected to double by 2040 as compared to 2008 [3]. Such rapid growth of the aging population that is ubiquitous and never seen in the history of civilization is linked to an increase in mental disorders among older adults [4, 5]. Further, depression is mostly portrayed by loss of interest in day-to-day activities, loss/gain in weight, sleeping disorder, and feelings of guilt and worthlessness, leading to impairments in one’s functional ability, accompanied by other diseases consequently deteriorating their quality of life [6, 7], indicating that mental health disorder is directly associated with the physical health and disability.

In most of the developing countries including India, globalization led to changes such as rapid urbanization, and out-migration of younger adults that are associated with diminishing preference for intergenerational co-residence further resulting in a tremendous psychological impact on the well-being of the senior citizens/older adults [8–12]. However, the severity of psychological performance or wellbeing, among older adults varies from person to person, older adults in India face a horde of psychological problems. Depressive symptoms mostly resulting from different socioeconomic factors as well as low spiritual intelligence and disturbed sleep are the most common among all [5, 13, 14]. Older adults in India possesses unique cultural characteristics where their vulnerability in terms of educational and occupational status and economic dependency is closely associated with their poor self-rated health respectively [15] However, their perceived health deteriorates with age and the effect of ill-health is closely related to poor psychological wellbeing [16]. An unhealthy lifestyle and negligence towards the physical activity in old age increases the burden of psychological distress among the older adults in India [17]**.**

It is well-known that low socioeconomic status (SES) is inter-related with high psychiatric morbidity and mental health situations. People with weak social support are prone to have a higher prevalence of psychological distress [13, 18]. Globally, evidence from developed and developing countries has encountered that people coming from lower wealth gradient are more vulnerable to have stressful life events further adding more risk for common mental health disorders and psychological distress situations [19]. There also exists inequality in the prevalence of mental health disorders across different socioeconomic groups within a population. A study in India indicates a higher prevalence of depression among the poorest and the uneducated men and women [20]. Furthermore, since the distribution of risk exposure is non-random, some population strata within societies are more likely to have higher psychological distress due to their vulnerability to poor social, economic, and environmental factors, correlated with gender [21]. Previous studies evidenced the presence of a positive relationship between income inequality and risk of psychological disorder, however, poor psychological health was found to be more common among older adults belonging to economically backward class. Psychological distress which is captured in the form of mental health disorder or depression is inversely proportional to the socio-economic status of the household, respectively [22–24]. Apart from this, there also lies a significant association between poverty and psychological distress identified in the form of common mental health disorders in low-income countries [25].

Older adults with less financial support are more prone to be socially isolated (reduction in social roles) and have a negative effect on their mental/physical health conditions, further depicting a link between income and health respectively [26]. Studies from China and Japan suggest that living arrangements and marital status of older adults are found to have statistically significant implications on mental health and well-being, more specifically women living alone or with a physical disability had higher chances of suffering from depressive symptoms [27, 28]. Few studies from India and China reveal that older adults with larger friend circles had better psychological health than their counterparts, and those living alone were at higher risk of having depressive symptoms and psychological distress [29, 30]. However, another Indian study found that the psychological distress of older adults was not significantly associated with marital status and familial structures, though it could be possibly due to the financial independence of the older adults [12]. Moreover, violence against older adults being a stressful event has been found to have significant psychological consequences such as depression and anxiety [31–33]. A study in India suggests that the chances of suffering from psychological distress among older adults are higher among those who have faced any type of violence more recently as compared to their counterparts with no such experiences [34].

The interrelation between socioeconomic and health status and inequality in psychological distress of the older population is an area of research that is yet to receive the desired attention in India. Previous studies have generated evidence that lower SES affects the health condition of the population in general [35]. However, there is a gap in literature focusing on socioeconomic and health status and its effect on inequality in psychological distress among older adults in India. Hence, the present study aims to add to the literature on the associations of socioeconomic and health status of older adults and their psychological distress. Further, the study hypothesized that.

H_1_: There is a negative relationship between the socio-economic condition and psychological distress among older adults.

H_2_: There is a concentration of psychological distress among older adults from households with poor economic status.

## Methods

### Data

The present research used data from Building a Knowledge Base on Population Ageing in India BKPAI, which is a nationally representative survey and was conducted in 2011, across seven states of India [36]. It was sponsored by Institute for Social and Economic Change (ISEC), Tata Institute for Social Sciences (TISS), Institute for Economic Growth (IEG), New Delhi, and United Nations Population Fund (UNFPA). The survey gathered information on various socio-economic and health aspects of the aging population from households of those aged 60 years and above. Seven major regionally representative states were selected for the survey with the highest 60+ years. This survey was carried out on a representative sample in the northern, western, eastern, and southern parts of India following a random sampling process [36].

The primary sampling unit (PSU) were villages for rural areas and urban wards in urban areas. The sample of 1280 elderly households was fixed for each state. Further details on the sampling procedure, the sample size is available in national and state reports of BKPAI, 2011. For the current study, a sample size of 9540 older adults residing in seven states aged 60+ years was selected. The urban and rural samples within each state were drawn separately. The PSUs in the rural areas were villages, whereas the urban wards were the PSUs in the urban areas. First, villages were classified into different strata based on population size, and the number of PSUs to be selected was determined in proportion to the population size of each stratum. Using probability proportional to population size (PPS) technique, the PSUs were selected and within each selected PSU, elderly households were selected through systematic sampling. A similar procedure was applied in drawing samples from urban areas.

Of a total of 10,604 elderly identified from 8329 household interviews, 9852 elderly interviews were completed either independently or through proxy interviews. The individual completion rate, which is the number of completed interviews (either independently or through a proxy) per 100 eligible elderly identified in the household, was 93%. The individual survey response rate for the states ranged from a low of 90% in Kerala and Punjab to a high of 98% in Tamil Nadu. To provide reliable measures sampling weights were generated at household and individual levels separately for rural and urban areas. Later, the design weight was calculated by adjusting for non-response at both the household and individual levels. The sample weights were further normalized at the state level to obtain standard state weights for each of the seven states so that the total number of weighted cases becomes equal to the total number of unweighted cases. The effective sample size was 9181 older adults. There were 671 missing cases in the data set.

### Variable description

#### Outcome variable

1. Psychological distress was measured from General Health Questionnaire (GHQ-12). The value of Cronbach alpha was 0.90. It was having a scale of 0 to 12 on the basis of experiencing stressful symptoms and was re-coded as 0 (representing 6+ stressful symptoms) and 1 (representing 5 and fewer symptoms) [20, 37]. The variable was coded using 12 questions namely a. Recently able to concentrate on whatever doing b. Recently lost much sleep due to some worry c. Recently felt constantly under strain d. Recently felt like couldn’t overcome difficulties e. Recently been feeling unhappy and depressed f. Recently been losing self-confidence g. Recently been thinking self as a worthless person h. Recently felt like playing a useful role in life i. Recently felt capable of making decisions about things j. Recently been able to enjoy normal day-to-day activities k. Recently been able to face up problems l. Recently been feeling reasonably happy, all things considered.

#### Control variables

The control variables were included in the study as per the literature reviewed [38–40]. Age was categorized as 60–69, 70–79, and 80+ years. Gender was categorized as Men and Women. Religion was categorized as Hindu, Muslim, Sikh, and others. Caste was categorized as Scheduled Caste, Scheduled Tribes, Other Backward Class, and others [41]. Place of Residence was categorized as rural and urban. Educational status was categorized as no education, below 5 years of schooling, 6–10 years of schooling, and 11 and above years of schooling. Marital status was categorized as not in union “included never married, widowed, divorced and separated” and currently in the union. The wealth index drawn from the BKPAI survey is based on the following 30 assets and housing characteristics: household electrification; drinking water source; type of toilet facility; type of house; cooking fuel; house ownership; ownership of a bank or post-office account; and ownership of a mattress, a pressure cooker, a chair, a cot/bed, a table, an electric fan, a radio/transistor, a black and white television, a color television, a sewing machine, a mobile telephone, any landline phone, a computer, internet facility; a refrigerator, a watch or clock, a bicycle, a motorcycle or scooter, an animal-drawn cart, a car, a water pump, a thresher, and a tractor. The range of index was from poorest to the richest i.e. ranging from lowest to the highest [36].

Source of income was categorized as 0 “no income” 1 “one source of income” and 2+ “two or more source of income”. Working status for the last year was categorized as no and yes. Five questions for involvement in the community were asked and were used to create a variable to measure social capital. The score developed range from 0 to 5, and a score of 0 was categorized as 0 “no community involvement” and a score of 1 to 5 was categorized as 1 representing any personal involvement in the community. ‘How important do you feel your presence in the family?’ was categorized as “important” and “somewhat or not important”. Violence against older adults was categorized to ‘no’ as “no violence” and ‘yes’ as “older adult experienced violence”. The variable was a combination of violence/abuse/neglect, the response was coded to 0 as “no violence” if the respondent didn’t face any type of violence and to 1 as “yes” if the respondent faced violence or abuse or neglect. Multi-morbidity was counted from dichotomous responses of 20 chronic morbidities asked the participants. It was re-coded as 0 representing “no morbidity”, 1 “having single morbidity” and 2+ “having two or more morbidity”. Disability was re-coded to 0 as it represents “no disability”, 1 “having one disability” and 2+ “having two or more disability”.

Ability to do activities of daily living was having a scale of 0 to 6 wherein it represents higher the score higher the independence. A score was categorized as 0, which represents full independence and 5 and less was categorized as 1, which represents not fully independent to do activities of daily living (Cronbach alpha: 0.93). The ability to do instrumental activities of daily living was having a scale of 0 to 8, representing higher the score higher the independence. A score of 6+ was categorized as 0 representing high IADL and a score of 5 and less was recoded as 1 representing low IADL [42–44]. Cognitive ability was measured by the number of words recalled. To measure cognitive ability, a scale of 0 to 8 was prepared, representing higher the score better the cognitive ability. Five or more words were recorded as “0” representing better cognitive ability and a score of four or less was recorded as “1” representing low cognitive ability [45, 46].

### Statistical analysis

Descriptive statistics were used to show the distribution of the study population. A Multi-collinearity test was conducted before the multivariate analysis [47] and it was evident that there no multicollinearity present in the data set. Further, bivariate and multivariate analysis was used to identify the factors associated with the outcome variable. The svyset command was used in STATA 14 [48] to account for complex survey design [49]. Additionally, survey weights were used to provide the weighted estimates for the outcome variable in the present study.

The study used the wealth quintile for decomposition analysis and the calculation of Concentration Index (CCI), the wealth quintile status used, was divided into five equal sizes of the population [50].

### Concentration index

Concentration index presents the magnitude of inequality by measuring the area between the concentration curve and line of equality and is calculated as twice the weighted covariance between the outcome and fractional rank in the wealth distribution divided by the variable mean [41, 50, 51].

The concentration index can be written as follows:

$$ C=\frac{2}{\mu}\mathit{\operatorname{cov}}\left({y}_{i,}{R}_i\right) $$

Where C is the concentration index; *y*_*i*_ is the outcome variable index; *R* is the fractional rank of individual *i* in the distribution of socio-economic position; *μ* is the mean of the outcome variable of the sample, and *cov* denotes the covariance [52]. The index value lies between − 1 to + 1.

Further, the study decomposes the concentration index to understand the relative contribution of various socio-economic factors to the psychological distress among older adults. To do this, the study used a regression-based decomposition technique, which was proposed by Wagstaff et al. [53]. In this model, psychological distress among older adults is considered the outcome variable for assessing the effect of SES on inequalities.

## Results

Table 1 represented the percentage distribution of background characteristics among the target population in the study. The percentage of older adults with psychological distress was around 23.5%.

**Table 1:** Percentage distribution of background characteristics among older adults in India (*N* = 9181)

Variables	N	Percentage
**Psychological distress**		
Low	7027	76.5
High	2154	23.5
**Age (years)**		
60–69	5815	63.3
70–79	2437	26.5
80+	929	10.1
**Gender**		
Men	4353	47.4
Women	4828	52.6
**Religion**		
Hindu	7389	80.5
Muslim	617	6.7
Sikh	771	8.4
Others	404	4.4
**Caste**		
Scheduled Caste	1802	19.6
Scheduled Tribe	470	5.1
Other Backward Class	3190	34.8
Others	3719	40.5
**Place of residence**		
Rural	4784	52.1
Urban	4397	47.9
**Educational status**		
No education	4186	45.6
Below 5 years	1886	20.5
6–10 years	2310	25.2
11+ years	799	8.7
**Marital status**		
Not in union	3724	40.6
Currently in union	5457	59.4
**Wealth**		
Poorest	1758	19.2
Poorer	1838	20.0
Middle	1823	19.9
Richer	1844	20.1
Richest	1918	20.9
**Source of income**		
0 “No source”	3940	42.9
1	4318	47.0
2+	923	10.1
**Working status**		
No	7076	77.1
Yes	2105	22.9
**Community involvement**		
No	1753	19.1
Yes	7428	80.9
**Living arrangement**		
Alone	556	6.1
With spouse	1339	14.6
Others	7286	79.4
**How important do you feel you are important to your family?**		
Important	5987	65.2
Somewhat or not important	3194	34.8
**Violence**		
No	8280	90.2
Yes	901	9.8
**Multi-morbidity**		
0 “No morbidity”	3260	35.5
1	2936	32.0
2+	2985	32.5
**Disability**		
0 “No disability”	2485	27.1
1	2816	30.7
2+	3880	42.3
**ADL**		
High “6+ score”	8521	92.8
Low “5 or less score”	660	7.2
**IADL**		
High “6+ score”	4276	46.6
Low “5 or less score”	4905	53.4
**Cognitive ability**		
High *“scores 5+”*	3888	42.4
Low *“scores of 4 or less”*	5293	57.7
**State**		
Kerala	1338	14.6
Himachal Pradesh	1452	15.8
Punjab	1249	13.6
West Bengal	1112	12.1
Orissa	1448	15.8
Maharashtra	1251	13.6
Tamil Nadu	1331	14.5
**Total**	9181	100.0

N: Sample

The mean distribution of Psychological distress over the background characteristics of the target population in this study were summarized in Table 2. Findings from this table showed that with the increase in age, psychological health deteriorated since a higher mean score over this table resembled high psychological distress. As compared to men, women had a higher mean score for psychological distress. The mean score for psychological distress was also higher for older adults living alone, which indicated that older adults living with their spouse only or with others were at a better place than the ones left with no one. Older adults, who felt that they were only somewhat or not at all necessary for their family members, had a higher degree of psychological distress. Likewise, older adults who had experienced physical/mental violence reported having low psychological as compared to the counterpart. Findings from this table also suggested that older adults with a higher number of multi-morbidity and disabilities had greater psychological distress. Older adults with low ADL, low IADL, and low cognitive ability were at the worst state of psychological health with higher mean scores.

**Table 2:** Mean distribution of psychological distress over background characteristics among older adults in India (*N* = 9181)

Variables	Mean	CI (95%)
**Age (years)**		
60–69	0.20	0.205–0.195
70–79	0.27	0.279–0.261
80+	0.35	0.366–0.334
**Gender**		
Men	0.21	0.216–0.204
Women	0.26	0.266–0.254
**Religion**		
Hindu	0.26	0.265–0.255
Muslim	0.23	0.247–0.213
Sikh	0.08	0.090–0.070
Others	0.16	0.178–0.142
**Caste**		
Scheduled Caste	0.28	0.291–0.269
Scheduled Tribe	0.32	0.342–0.298
Other Backward Class	0.26	0.268–0.252
Others	0.17	0.176–0.164
**Place of residence**		
Rural	0.25	0.256–0.244
Urban	0.19	0.196–0.184
**Educational status**		
No education	0.31	0.317–0.303
Below 5 years	0.22	0.230–0.210
6–10 years	0.13	0.137–0.123
11+ years	0.08	0.090–0.070
**Marital status**		
Not in union	0.29	0.297–0.283
Currently in union	0.20	0.205–0.195
**Wealth**		
Poorest	0.37	0.382–0.358
Poorer	0.29	0.301–0.279
Middle	0.20	0.209–0.191
Richer	0.15	0.158–0.142
Richest	0.09	0.097–0.083
**Source of income**		
0 “No source”	0.27	0.277–0.263
1	0.22	0.226–0.214
2+	0.17	0.182–0.158
**Working status**		
No	0.25	0.255–0.245
Yes	0.25	0.259–0.241
**Community involvement**		
No	0.35	0.361–0.339
Yes	0.21	0.215–0.205
**Living arrangement**		
Alone	0.33	0.350–0.310
With spouse	0.21	0.221–0.199
Others	0.23	0.235–0.225
**How important do you feel you are important to your family?**		
Important	0.16	0.165–0.155
Somewhat or not important	0.36	0.369–0.351
**Violence**		
No	0.22	0.225–0.215
Yes	0.34	0.356–0.324
**Multi-morbidity**		
0 “No morbidity”	0.20	0.207–0.193
1	0.25	0.258–0.242
2+	0.26	0.268–0.252
**Disability**		
0 “No disability”	0.12	0.127–0.113
1	0.18	0.187–0.173
2+	0.35	0.358–0.342
**ADL**		
High “6+ score”	0.21	0.214–0.206
Low “5 or less score”	0.51	0.529–0.491
**IADL**		
High “6+ score”	0.14	0.145–0.135
Low “5 or less score”	0.30	0.307–0.293
**Cognitive ability**		
High *“scores 5+”*	0.14	0.146–0.134
Low *“scores of 4 or less”*	0.30	0.306–0.294
**State**		
Kerala	0.14	0.149–0.131
Himachal Pradesh	0.17	0.180–0.160
Punjab	0.07	0.077–0.063
West Bengal	0.29	0.304–0.276
Orissa	0.37	0.383–0.357
Maharashtra	0.23	0.242–0.218
Tamil Nadu	0.36	0.373–0.347
**Total**	0.23	0.234–0.226

*CI* Confidence Interval

Table 3 represented Model I and Model II, where the former gives the adjusted ORs for the relationship between wealth index/standard of living of the older adults, their source of income, their working status, and their psychological distress, after controlling for the background characteristics. In contrast, the latter gives the unadjusted ORs with all the background characteristics. Findings from the model I suggested that poorer older adults were 32% less likely to have greater psychological distress than the poorest ones. While the richer and the richest, on the other hand, were also less likely to have psychological distress as compared to the poorest one, which has remained significant [UOR:0.26; CI:0.22–0.31; *p*-value: 0.001and UOR:0.15; CI: 0.12 ~ 0.18; p-value: 0.001] in the model.

**Table 3:** Odds Ratio estimates for high psychological distress by background characteristics among older adults in India (*N* = 9181)

Variables	Model-I		Model-II	
	Unadjusted OR (95% CI)	***p***-value	Adjusted OR (95% CI)	***p***-value
**Age (years)**				
60–69			Ref.	
70–79			0.97(0.83,1.15)	0.760
80+			1.03(0.80,1.31)	0.828
**Gender**				
Men			Ref.	
Women			0.88(0.74,1.05)	0.164
**Religion**				
Hindu			Ref.	
Muslim			1.20(0.91,1.58)	0.197
Sikh			0.92(0.55,1.53)	0.737
Others			0.96(0.68,1.37)	0.833
**Caste**				
Scheduled Caste			Ref.	
Scheduled Tribe			0.87(0.64,1.16)	0.343
Other Backward Class			0.77(0.62,0.95)	0.014
Others			0.79(0.65,0.97)	0.026
**Place of residence**				
Rural			Ref.	
Urban			0.97(0.83,1.13)	0.675
**Educational status**				
No education			Ref.	
Below 5 years			0.77(0.64,0.93)	0.005
6–10 years			0.61(0.49,0.78)	0.001
11+ years			0.56(0.37,0.86)	0.008
**Marital status**				
Not in union			Ref.	
Currently in union			1.05(0.88,1.25)	0.587
**Wealth**				
Poorest	Ref.		Ref.	
Poorer	0.68(0.59,0.78)	0.001	1.10(0.91,1.34)	0.311
Middle	0.40(0.34,0.46)	0.001	0.87(0.69,1.09)	0.229
Richer	0.26(0.22,0.31)	0.001	0.83(0.64,1.08)	0.158
Richest	0.15(0.12,0.18)	0.001	0.60(0.43,0.83)	0.002
**Source of income**				
0 “No source”	Ref.		Ref.	
1	0.76(0.68,0.86)	0.001	1.01(0.85,1.20)	0.899
2+	0.54(0.44,0.68)	0.001	0.84(0.62,1.13)	0.246
**Working status**				
No	Ref.		Ref.	
Yes	0.73(0.63,0.85)	0.001	0.98(0.79,1.21)	0.825
**Community involvement**				
No			Ref.	
Yes			0.81(0.69,0.96)	0.014
**Living arrangement**				
Alone			Ref.	
With souse			0.71(0.50,1.00)	0.048
Others			0.83(0.63,1.11)	0.210
**How important do you feel you are important to your family?**				
Important			Ref.	
Somewhat or not important			1.81(1.56,2.09)	0.001
**Violence**				
No			Ref.	
Yes			1.78(1.43,2.22)	0.001
**Multi-morbidity**				
0 “No morbidity”			Ref.	
1			1.22(1.03,1.45)	0.024
2+			1.47(1.21,1.77)	0.001
**Disability**				
0 “No disability”			Ref.	
1			1.79(1.44,2.21)	0.001
2+			3.49(2.82,4.32)	0.001
**ADL**				
High “6+ score”			Ref.	
Low “5 or less score”			1.92(1.50,2.46)	0.001
**IADL**				
High “6+ score”			Ref.	
Low “5 or less score”			1.38(1.18,1.62)	0.001
**Cognitive ability**				
High *“scores 5+”*			Ref.	
Low *“scores of 4 or less”*			1.76(1.50,2.08)	0.001
**State**				
Kerala			Ref.	
Himachal Pradesh			0.99(0.70,1.39)	0.943
Punjab			0.33(0.21,0.54)	0.001
West Bengal			1.34(1.01,1.78)	0.045
Orissa			2.27(1.69,3.04)	0.001
Maharashtra			1.42(1.07,1.87)	0.015
Tamil Nadu			6.03(4.47,8.13)	0.001

*Ref* Reference, *CI* Confidence Interval

Considering the source of income, findings suggested that, older adults with ‘one’ source of income were 24% [UOR: 0.76; CI: 0.68–0.86; p-value: 0.001] less likely to have psychological distress, and the one with ‘two or more” source of income were 46% [UOR: 0.54; CI: 0.0.44–0.68; p-value: 0.001] less likely to have the same as compared to the one with no source of income, which was even statistically significant with p-value< 0.05. Likewise, older adults who were working for the last 1 year (till the date of the survey) were less likely to have psychological distress than the one who was not working, and it had remained significant [UOR:0.73; CI:0.63–0.85, p-value: 0.001] in the model. Findings from Model II suggested that caste, wealth index, educational status, community involvement, living arrangements, familial importance, multi-morbidity, disability, ADL, IADL, cognitive ability, and states were statistically and significantly associated with psychological distress with p-value< 0.05.

**Fig. 1:**
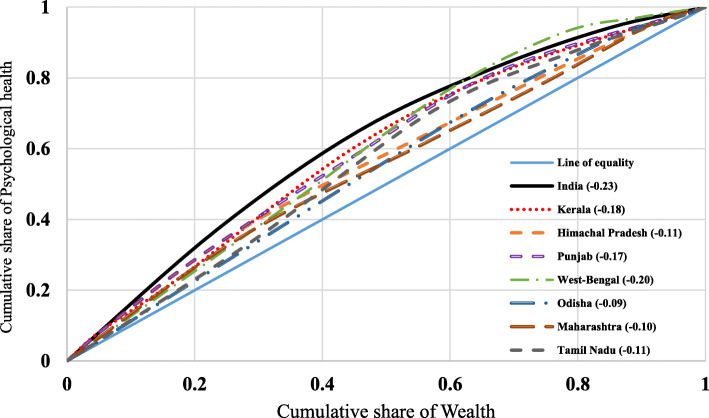
Concentration curve for low psychological distress among older adults in India and selected states

Figure 1 depicted the concentration curve for psychological distress among older adults in India and selected states. The concentration curve for India lies above (dominates) the line of equality, indicating that greater psychological distress was concentrated among the poor. This figure also indicated that, among all the selected states, Odisha had less inequality in psychological distress. In contrast, it was higher in Kerala, though less than the national average (the line representing India dominated all the state curves).

Table 4 represented results from the decomposition analysis and showed how the various background characteristics of respondents viz. age, gender, religion, caste, place of residence, education, marital status, source of income, working status, community involvement, living arrangement, familial importance, violence, multi-morbidity, disability, ADL, IADL, and cognitive ability, contributed to the economic inequality in psychological distress. The concentration index was given both in terms of absolute (same units as the concentration index) and percent contribution (adjusted percentage contribution of inequality). Findings from Table 4 suggested that educational status, familial importance, living arrangement, religion, caste, and cognitive ability were the significant contributors to the inequalities. Educational status was responsible for around 20% of the inequality in psychological distress among older adults, followed by familial importance responsible for about 15.6% of the inequality. Apart from this, religion, caste, and living arrangements also made a substantial contribution to the inequalities in psychological distress, explaining 14.0%, 11.6%, and 12.8% of the total inequality respectively.

**Table 4:** Estimates of decomposition analysis for the contribution of selected background factors to economic inequality to the high psychological distress among older adults in India (N = 9181)

Variables	Elasticity	CCI	Absolute contribution to CCI	Percentage Contribution to CCI
Age	0.024	0.001	0.000	0.1
Gender	0.062	−0.011	−0.001	−1.8
Religion	0.068	0.076	0.005	14.0
Caste	0.062	0.069	0.004	11.6
Place of residence	−0.007	0.052	0.000	−1.0
Educational status	0.059	0.125	0.007	20.0
Marital status	0.015	0.039	0.001	1.6
Source of income	0.024	−0.023	−0.001	−1.5
Working status	−0.003	−0.176	0.001	1.4
Community involvement	0.046	0.035	0.002	4.4
Living arrangement	0.118	0.040	0.005	12.8
How important do you feel you are important to your family?	−0.040	−0.144	0.006	15.6
Violence	−0.009	−0.192	0.002	4.7
Multi-morbidity	0.003	0.078	0.000	0.6
Disability	−0.096	−0.018	0.002	4.7
ADL	−0.012	−0.011	0.000	0.4
IADL	−0.024	−0.062	0.001	4.0
Cognitive ability	−0.039	−0.081	0.003	8.6
**Explained CCI**			0.037	100.0
**Actual CCI**			−0.233	
**Residual CCI**			−0.270	

*CCI* Concentration Index

## Discussion

The current study investigated the relationships between SES and inequality in psychological distress among older adults using nationally representative data from BKPAI. The study identified 23.5% of the older population in India as experiencing higher levels of psychological distress. Other epidemiological data about older Indian adults show that the prevalence of mental illnesses ranges from 2.2% to 61.2% [54–57]. Further, most of the SES indicators in the present study were significantly associated with psychological distress in later years. Also, huge geographical variations showed a regional impact of SES on inequality in psychological distress.

We found a negative association of gender, source of income, working status, as measured by the concentration index, with older people’s psychological distress. In our study, older people having no education were associated with greater psychological distress. This finding has been reported in many studies, particularly in developing countries that show a significant association of low educational status with a psychological disorder like depression has been reported [58–62]. Thus, our findings provide evidence that SES is a significant predictor of inequality in psychological distress [12]. The plausible reason would be that the older adults with lower SES were not able to get treatment for their poor mental health status or psychological distress. Globally, studies also indicate that the variables associated with improved mental and physical health of older adults include getting more income or wealth, more years of education, and a prominent career, as well as living in secure and safe communities [63–66].

Comparing different socio-economic groups concerning their sensitivity to socio-economic disparity shows that SES and psychological distress were negatively related. The middle and rich socioeconomic groups were less likely to have greater psychological distress compared to the poorest ones. In contrast, the respondents with no source of income had more chances of psychological distress. The finding indicates that a large proportion of this sub-population reported increased worries when incomes were more unequally distributed. Studies have shown that unemployment can also lead to reduced hope and financial difficulties, which in turn contribute to psychological disorders like depression [9, 67–69]. However, the higher concerns in the current study were particularly from the older population working for less than a year and had higher chances of having psychological distress.

Furthermore, we found that poor SES causes more psychological problems among those experiencing multi-morbidity, disability, ADL, IADL, and cognitive ability. A study documented that diabetes, low vision, and diabetes with hypertension have shown a substantial association with depression. Though hypertension alone is not associated with psychological distress [12, 70]. Diabetes and low vision interfering with daily routine activities (ADL) are related to the psychological state of depression [12, 71, 72]. Besides education, religion, and caste, family importance and living arrangements were the main contributors to inequality. A study documented that cultural norms can very well have an impact on the psychological outcomes, for example, the high societal value of care can lead to pride, and less burden (better psychological outcomes) or social pressure due to cultural expectations may require prolonged care and lead to psychological distress [29, 73].

The United Nations has emphasized the need for increased attention in its 17 sustainable development goals to factors that relate to the effect of socio-economic conditions on inequalities in health, including education, inclusion in policy decisions, employment, jobs, and differential SES. Thus, efforts should be made to address the true causes of inequality in psychological distress, and they may vary by region, culture, and gender, as illustrated [3].

The study has certain limitations. Since the present study was a cross-sectional study in which there was no thorough analysis of the relationship between causative factors for psychological distress, the reverse interpretation can also be possible. Again, the information on health outcomes was based on self-reporting, which may have led to under or over-estimation in the explanation of SES differences in psychological distress. However, the data was the best available to analyze the potential associations between SES and health-related variables and psychological distress among the older population in India.

### Study implications

Unlike other previous research on the association of socioeconomic and health-related variables with psychological distress, this study has clearly shown how socioeconomic and health conditions are associated with inequality in psychological distress among older adults.

Given the large proportion of psychological distress among the older age groups and the increasing size of the aging population, this study highlights the need for improving health care services and social security programs which can prevent the potential problems that older adults may encounter as they grow older. In general, improving older people’s health would, in turn, reduce government spending on health care needs. Also, more emphasis needs to be placed on the challenges of aging, and precisely the psychological health problems of aging (mental, emotional, social, and spiritual well-being), in terms of successful management and treatment of late-life psychological disorders.

The increased burden among specific sub-populations also highlights the importance of understanding the wider consequences of psychological health issues in older age groups, and how this puts additional pressure on those in the society who are already at a disadvantage. A better understanding of the psychosocial and physiological dynamics that underlie mental health inequalities among older adults can be an important step in helping to improve the overall wellbeing of an aging population. The results also reveal the policy challenges to prevent such inequalities, which may appear much earlier in the lives of people. Since the households and family members provide greater support in the old age in developing countries like India, they should be complemented by public policy that focuses on reducing poverty especially among the disadvantaged and those with low-income and from rural areas. The rapid population aging in such countries underlines the urgency of addressing the issues related to the older population.

## Conclusion

This study examined the psychological health status of an older population in India and found a large number of them reporting distress. There was some uncertainty regarding the magnitude of socioeconomic and health-related variables affecting inequality in psychological distress. This study suggests that among older people, there is a wide disparity of socio-cultural, demographic, and economic characteristics with significant factors responsible for inequality in psychological distress. Further, factors such as religion, caste, education, living arrangements, and self-worth in the family were major contributors to the concentration of psychological distress in older adults from poor households.

Preventive measures for psychological disorders need to be considered as an integral part of public health at local as well as national levels. The promotion of mental wellbeing should be incorporated into a public policy strategy encompassing horizontal intervention across numerous public sectors, such as social welfare, employment, education, health, and human rights. This will build “win-win” circumstances across sectors, including a broad spectrum of health, social and economic benefits. Future studies may investigate the causal associations between socioeconomic variables and the outcomes of mental health and inequalities. It is also hoped that further research may explore the relationship between personal or cultural attitudes towards mental health inequality among the older population.

## Data Availability

The study utilizes a secondary data which is available only on request from director@isec.ac.in or india.office@unfpa.org. The questionnaire and datasets generated and analysed during the current study are also available in the institute repository and accessible on request through http://www.isec.ac.in/

## Abbreviations

UOR: Unadjusted odd ratio
AOR: Adjusted odds ratio
CI: Confidence interval
CCI: Concentration Index
GHQ: General Health Questionnaire
BKPAI: Building a Knowledge Base on Population Ageing
PSU: Primary Sampling Unit
SES: Socio-economic status
ADL: Activities of daily living
IADL: Instrumental activities of daily living
