# Correction to: Socioeconomic inequality in psychological distress among older adults in India: a decomposition analysis

**DOI:** 10.1186/s12888-021-03231-0

**Published:** 2021-04-29

**Authors:** Shobhit Srivastava, Naina Purkayastha, Himanshu Chaurasia, T. Muhammad

**Affiliations:** 1 International Institute for Population Sciences, Mumbai, Maharashtra 400088 India; 2 Department of Statistics, Dibrugarh University, Dibrugarh, Assam India; 3 National Institute for Research in Reproductive Health, ICMR, Mumbai, 400088 India

**Correction to: BMC Psychiatry 21, 179 (2021)**

**https://doi.org/10.1186/s12888-021-03192-4**

Following the publication of the original article [1], an error was identified in the Results section of the Abstract. The updated section is given below and the changes have been highlighted in **bold typeface**.

Abstract:

*Results*

Older adults from the poorest **wealth quintile, having no source of income, not working for the last one year period, suffering from** multi-morbidity, disabled, with low activities of daily living and low instrumental activities of daily living and poor cognitive ability were suffering from high psychological distress in India. Further, factors such as religion, caste, education, living arrangements, and self-worth in the family were major contributors to the concentration of psychological distress in older adults from poor households (concentration index: − 0.23).
